# The Insurance Act Drug Tariff: Drastic Changes Proposed

**Published:** 1915-09-25

**Authors:** 


### THE INSURANCE ACT DRUG TARIFF.

### Drastic Changes Proposed.

The drug tariff which has been in operation since the Insurance Act came into force was prepared at a time of great urgency to meet circumstances which could not be foreseen, and as to which no guidance was afforded by previous experience. Three years' operation of that tariff has discovered many faults both in detail and principle, and it would have been surprising if the Departmental Committee which was appointed early in the present year to consider the matter had not declared the tariff to be based on an unscientific foundation. Under existing conditions every ingredient in a prescription is charged for, and, with certain exceptions, there is a dispensing fee in respect of the prescription as a whole. The charge for each ingredient comprises a profit at a varying ratio on the cost to the panel chemist of the actual quantity of the particular drug, the ingredient price being intended to afford the chemist a margin for "trade profit" or establishment charges. The intention of the framers of the tariff appears to have been that the chemist should look in the main to the dispensing fee for remuneration in respect of his personal service. In the opinion of the Committee, whose report has just been issued, the system of charging a profit calculated on each ingredient is not the best one, and they have come to the conclusion that the allowance for establishment charges which the chemist's remuneration must necessarily include should be conveyed to him in the form of a rate per prescription instead of a percentage upon the cost of drugs. Accordingly they recommend that a tariff providing for payment of (*a*) the cost price of the drugs supplied, (*b*) a flat rate per prescription for establishment expenses, and (*c*) a fee per prescription for any professional services, graded according to the nature of the prescription, should be adopted for the whole of Great Britain.

### The Panel Chemist's Point of View.

The figure fixed for the establishment charges is remarkably low, and there is little doubt that the sum of the charges for a hundred prescriptions under the proposed tariff would be very considerably less than under the existing arrangements for the same prescriptions. Other things being equal, therefore, the chemist would be a loser by the new terms. But there are two compensating factors recommended by the Committee; one is that the discounting clause in the chemists' agreement should be abolished, and the other that payment of the accounts should be reasonably prompt. At present the maximum amount available for the chemists' bills in any district is at the rate of two shillings per insured person; it is common knowledge that this sum has been hopelessly inadequate in many districts to pay the bills in full, and in consequence they have been subject to discounting which in some cases amounts to the high rate of 40 per cent. Moreover, there have been absurdly long delays in paying the accounts, with the consequence that those on the panel have suffered considerable hardship. The certainty of full and prompt payment may reconcile the majority of chemists to lower charges, but the adoption of the new tariff without these two factors would probably result in resignations from the panel.

### The Panel Doctor's Point of View.

The abolition of the discounting clause would mean that the sharp division between the *per capita* allowance for drugs and for medical attendance would disappear, and that the chemists' accounts would be the first charge on the Medical Benefit Fund. At first sight this proposal might appear detrimental to the interests of National Health Insurance practitioners. For instance, it might be argued that the prescriptions in an imaginary district would be so costly as to absorb the whole of the sum allotted for medical benefit. But whose fault would that be? Some months ago we warned the readers of The Hospital that if no other means could be found to check extravagance in prescribing the Commissioners might adopt some such expedient as is now proposed. We do not by any means suggest that the abnormal drain on the drug funds that has been so persistent in some districts is wholly due to over-prescribing, but it has been proved beyond a shadow of a doubt that gross extravagance has been practised, and when we issued the warning referred to above we pointed out that it was clearly in the interests of the profession to restrain such of their colleagues as were guilty of this practice. It will now be apparent to every panel doctor that any practitioner who prescribes unnecessarily costly drugs and in unnecessarily large quantities is damaging the interests of his brother insurance practitioners. We think that knowledge will be sufficient to check extravagance; and provided insurance prescribing is on a scale that is reasonable, and at the same time sufficient, we hope few, if any, cases will occur in which the amount available for paying the panel doctors' fees is less than it is at present.

### The Insured Person's Point of View.

In discussions of this kind the insured person is sometimes apt to be overlooked. What will be the position of the patient under the new tariff? The answer to this question is that it will be, if anything, better than it is at present. The chemists will be paid in full for all they dispense, and their contracts will include an obligation to supply a grade of drug costing them approximately the price allowed in the tariff. It has been the experience of the majority of insurance districts that an efficient pharmaceutical service can be provided for the figure originally allotted to pay for this service. Practitioners are not encouraged by the new proposals to practise economy to an unreasonable extent, and there is nothing to indicate that the patient is likely to obtain an inadequate supply of medicine. Looking at the proposals from all points of view, we can find few real objections to them; but it will be interesting to hear the opinion of panel doctors.

